# Supplementary material for: Self-sustained frictional cooling in active matter
Source: Nat Commun. 2025 Aug 6;16:7235. doi: 10.1038/s41467-025-62626-9 (PMC12328619; doi:10.1038/s41467-025-62626-9)
Supplement: Supplementary file 1 — Supplementary Information [file 41467_2025_62626_MOESM1_ESM.pdf]

Supplementary Information for
"Self-sustained frictional cooling in active matter"

Alexander P. Antonov,[1,*] Marco Musacchio,[1] Hartmut Löwen,[1] and Lorenzo Caprini[2,†]

[1] *Institut für Theoretische Physik II: Weiche Materie, Heinrich-Heine-Universität Düsseldorf, Universitätsstraße 1, D-40225 Düsseldorf, Germany*

[2] *Physics department, University of Rome La Sapienza, P.le Aldo Moro 5, IT-00185 Rome, Italy*

SUPPLEMENTARY DISCUSSION 1. HETEROGENEITY ANALYSIS FOR THE EXPERIMENTAL VIBROBOTS

In this Supplementary Discussion, we verify that particle and spatial heterogeneity play a negligible role in our experimental setup. To this end, we present additional experiments analyzing both spatial and particle heterogeneity. Specifically, we conduct single-particle experiments under the same conditions used to investigate collective phenomena. We measure the speed probability distribution $p(v)$ for 8 randomly selected particles, each moving independently on the vibrating plate for 5 minutes. As in the main text, particles located within one vibrobot diameter from a confining wall are excluded from the distribution analysis.

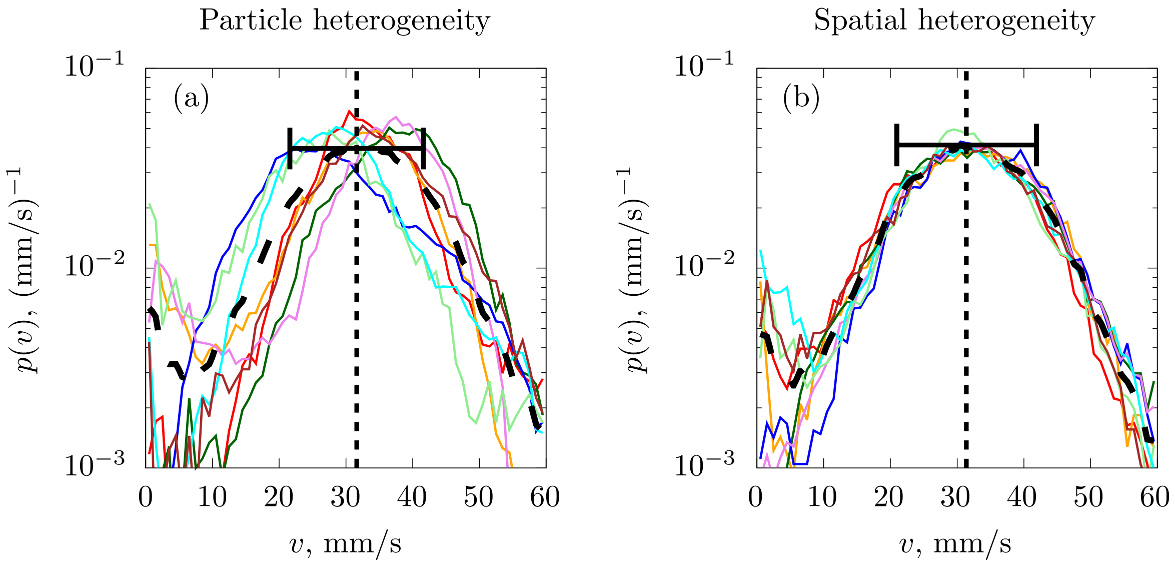

FIG. S1. **Particle and spatial heterogeneity.** (a) Speed probability distributions $p(v)$ for 8 randomly chosen particles denoted by different colors. (b) Speed probability distributions $p(v)$ measured for particles moving within 8 radial sectors calculated from the center of the arena. Distributions obtained in different spatial regions are differently colored. The two thick-dashed lines in (a) and (b) correspond to the average distribution, with mean and variance represented by a thin-dashed vertical line and the error bar, respectively.

Figure S1 (a) shows the speed probability distributions $p(v)$ for 8 individual particles randomly picked. In all the cases, the distributions exhibit Gaussian comparable profiles, confirming the negligibility of particle heterogeneity in our system. Figure S1 (b) displays $p(v)$ for a single particle conditioned to the particle position on the plate. In practice, we have divided the space into 8 radial sectors, each spanning 45°, and we have calculated $p(v)$ when the particle moves in that spatial region. In this case, the absence of spatial heterogeneity is evident, since $p(v)$ calculated in different regions (different colors) exhibits almost overlapped Gaussian profiles. In both cases, the typical speed

* alexander.antonov@hhu.de
† lorenzo.caprini@uniroma1.it

distribution shows a small peak near $v = 0$ reflecting the spontaneous change of direction (as observed in Ref. [1]), which leads to a local suppression of the particle translational velocity.

SUPPLEMENTARY DISCUSSION 2. SELF-SUSTAINED FRICTIONAL COOLING MECHANISM FOR PARTICLES INTERACTING THROUGH THE WEEKS-CHANDLER-ANDERSON (WCA) POTENTIAL.

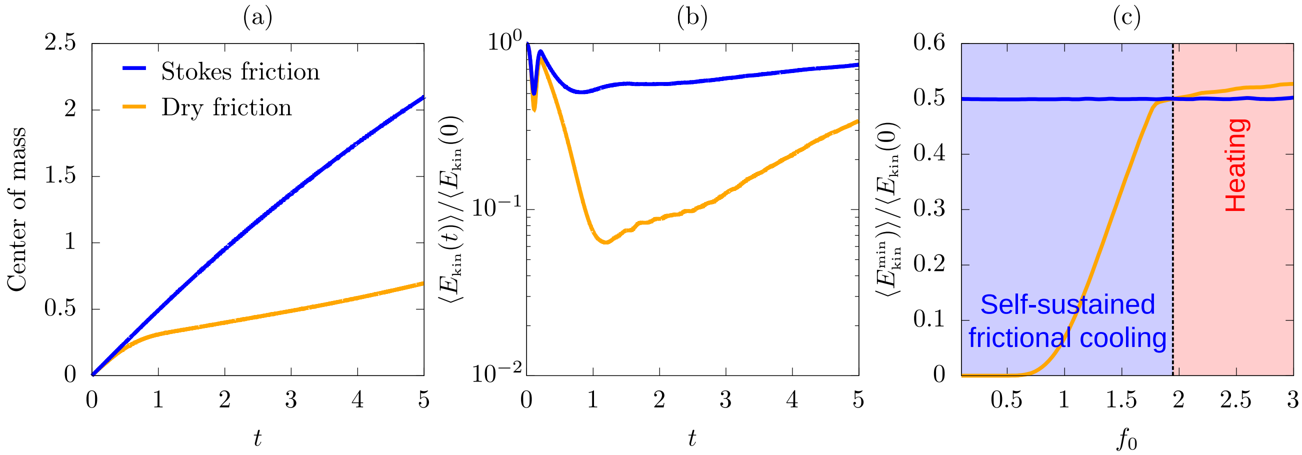

FIG. S2. **Collision between two active particles interacting through the WCA potential.** (a) Center of mass of two particles and (b) average mean kinetic energy as a function of time t. The kinetic energy is normalized by the initial activation energy $E_{\rm kin}(0) = mv_0^2/2$. Particles interact via exclusion-volume WCA potential, as in the numerical study reported in the main text. In both systems – with dry and Stokes friction – the kinetic energy initially drops to a minimum value, $E_{\rm min}^{\rm kin}$, due to exclusion volume interactions. This early stage is marked by non-monotonic variations in kinetic energy in panel (b) as the particles begin to interact. Subsequently, after reaching the minimum value, the system regains kinetic energy due to fluctuations in the active force. The recovery is generally slower in the case with dry friction compared to the system with Stokes friction, as shown in panel (a). (c) Minimal kinetic energy $E_{\rm min}^{\rm kin}$ during the cooling process as a function of activity f_0. For low activity, dry friction cools the system more effectively than Stokes friction, showing a transition from “Self-sustained frictional cooling” to “Heating”. The transition is indicated by a vertical dashed line, which marks the activity threshold beyond which dry friction becomes less effective than Stokes friction at dissipating energy. The dimensionless parameters of the simulations are $\tau_0^{-1} = 0.1$, $\epsilon_0 = 1$, $\gamma_0 = 1$, $d = 1$, and $f_0 = 1$ for panels (a) and (b).

In the main text, we report a proof-of-concept numerical study to evaluate the effect of dry friction on a collision between two active particles interacting through elastic hardcore interactions, i.e. the self-sustained frictional cooling. To show the generality of this mechanism, in this Supplementary Discussion, we repeat the proof-of-concept analysis for two colliding particles interacting through an exclusion volume Weeks-Chandler-Anderson potential employed in the numerical study (Figs. 3-5). Specifically, the system of 2 particles evolves with the following one-dimensional dynamics for the particle position x_i and the velocity v_i:

$$\dot{x}_i(t) = v_i(t), \tag{S1a}$$

$$\dot{v}_i(t) = -\sigma(v_i(t)) + \sqrt{\frac{2}{\tau_0}}\xi_i(t) + f_0 n_i + \sum_{j\neq i}\partial_{x_i} U_{\rm WCA}(|x_i - x_j|), \tag{S1b}$$

$$\dot{n}_i(t) = -\frac{n_i(t)}{\tau_0} + \sqrt{\frac{2}{\tau_0}}\eta_i(t)\,, \tag{S1c}$$

where $i = 1, 2$. The friction force $\sigma(v)$ is $\sigma(v) = {\rm sgn}(v)$ for dry friction, and $\sigma(v) = \gamma_0 v$ for Stokes friction, where $\gamma_0 = 1$ is the Stokes damping coefficient. The Weeks-Chandler-Andersen (WCA) potential $U_{\rm WCA}$ has the form

$$U_{\rm WCA}(|x_i - x_j|) = \begin{cases} 4\epsilon_0\left[\left(\frac{d}{x_i - x_j}\right)^{12} - \left(\frac{d}{x_i - x_j}\right)^{6}\right], & \text{if } |x_i - x_j| < 2^{1/6}d, \\ 0, & \text{else}\,. \end{cases} \tag{S2}$$

where d is the particle diameter. As in the numerical study reported in the main text, we have rescaled the time with $\sqrt{\tau K}/\Delta_C$, the length with $\tau K/m\Delta_C$, and the force with Δ_C. The dimensionless parameters governing the dynamics are the activity $f_0 = f/\Delta_C$, the reduced noise strength $1/\tau_0 = \sqrt{K/\tau}/\Delta_C$, and the reduced potential strength ϵ_0 are discussed in the Methods of the main text.

Equations (S1) are solved numerically using the EulerMaruyama scheme with a time step $\Delta t = 10^{-5}$, and simulation parameters $\tau_0^{-1} = 0.1$ and $d = 1$. The simulations are initialized by chosing the activated particle with velocity $v_1(0) = v_0 = f_0/\gamma_0$, activity $n_1(0) = 1$ and coordinate $x_1(0) = -2^{-5/6}d$, and the particle at rest with zero initial velocity and activity $v_2(0) = n_2(0) = 0$, and coordinate $x_2(0) = 2^{-5/6}d$ (i.e., the two particles are placed exactly at the contact distance defined by the WCA interaction). Figure S2 shows the dynamical evolution of the ensemble average computed over 10^3 stochastic trajectories. Figure S2 (a) plots the time trajectory of the center of mass of the system, while Fig. S2 (b) reports the time trajectory of the kinetic energy for $f_0 = 1$. Similarly to hardcore interactions, this study demonstrates that cooling by dry friction is stronger than the one obtained by Stokes friction, since the particles are slower on average and lose their kinetic energy much faster in the system with dry friction. By tracking the minimum kinetic energy during the cooling process (Fig. S2 (c)), we observe that dry friction leads to more effective cooling at low activity levels, whereas higher activity results in particle heating. These findings are consistent with our observations for hardcore elastic interactions reported in Fig. 2 of the main text. The softness of the WCA potential becomes relevant only at high activity levels f_0, where the distinction between Stokes friction and dry friction is much less pronounced than in systems with hard-core elastic interactions.

SUPPLEMENTARY DISCUSSION 3. SELF-SUSTAINED FRICTIONAL COOLING MECHANISM FOR PARTICLES WITH PARTIALLY INELASTIC COLLISIONS.

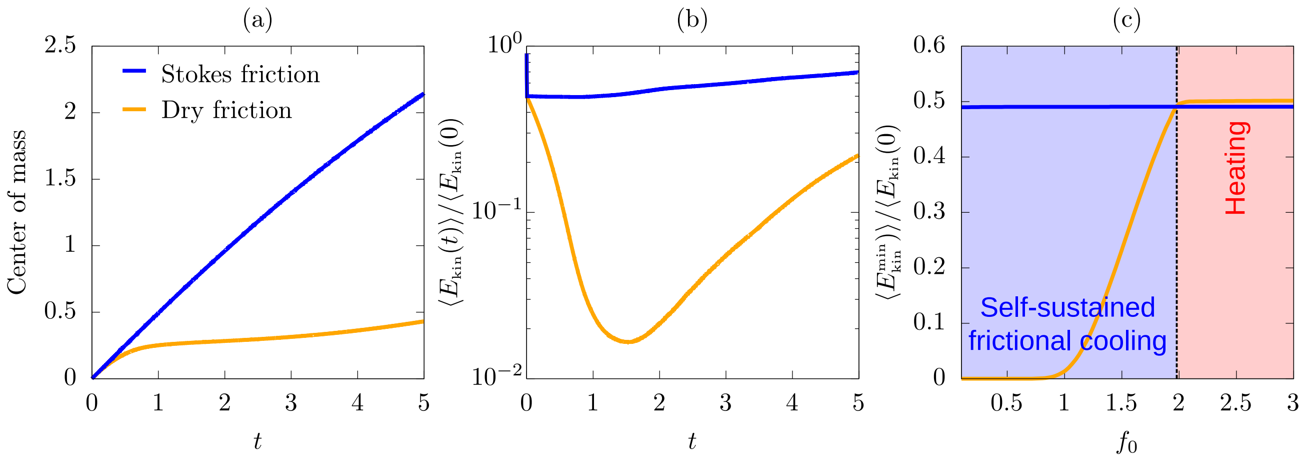

FIG. S3. **Inelastic collision between two active particles.** (a) Center of mass of two particles and (b) average mean kinetic energy as a function of time t. The kinetic energy is normalized by the initial activation energy $E_{\rm kin}(0) = mv_0^2/2$. (c) minimal kinetic energy $E^{\rm kin}_{\rm min}$ during the cooling process as a function of activity f_0. Similarly to both the elastic hard-core and WCA interactions, for low activity dry friction leads to more efficient cooling of the system compared to Stokes friction, revealing a transition from “Self-sustained frictional cooling” to “Heating”, marked by a dashed vertical line. The two active particles collide by following the hard-core inelastic collision rule, as described in the text (Eq. (S4)). The dimensionless parameters of the simulations are $\tau_0^{-1} = 0.1$, $\epsilon_0 = 1$, $\gamma_0 = 1$, $d = 1$, $\kappa = 0.9$, and $f_0 = 1$ for (a) and (b).

To further support the generality of the self-sustained frictional cooling discussed in the previous section, we consider the case of two particles undergoing partially inelastic collisions. Specifically, the system governed by dry friction

evolves with the following one-dimensional dynamics for the particle position x_i and the velocity v_i:

$$\dot{x}_i(t) = v_i(t), \tag{S3a}$$

$$\dot{v}_i(t) = -\sigma(v_i(t)) + \sqrt{\frac{2}{\tau_0}}\xi_i(t) + f_0 n_i, \tag{S3b}$$

$$\dot{n}_i(t) = -\frac{n_i(t)}{\tau_0} + \sqrt{\frac{2}{\tau_0}}\eta_i(t)\,, \tag{S3c}$$

where $i = 1, 2$. To include inelasticity, collisions are treated according to the following procedure:

i) Compute the collision time: $t_{\rm coll} = \dfrac{x_2 - x_1 - d}{v_1 - v_2}$, where d is the particle diameter.

ii) If $t_{\rm coll} \leq 0$ or $t_{\rm coll} \geq \Delta t$, proceed with the EulerMaruyama update for all particles without modification.

iii) Otherwise, update the particle positions using their initial velocities up to the collision time $t_{\rm coll}$.

iv) At the collision, update the velocities as

$$v_1' \leftarrow \frac{1}{2}\left[v_1(1+\kappa) + v_2(1-\kappa)\right], \quad v_2' \leftarrow \frac{1}{2}\left[v_1(1-\kappa) + v_2(1+\kappa)\right], \tag{S4}$$

where the prime denotes post-collisional velocities. The constant κ is the coefficient of restitution: $\kappa = 1$ corresponds to perfectly elastic collisions, as considered in the main text (Fig. 2 of the main text), while $\kappa = 0$ corresponds to perfectly inelastic collisions. In this study, we use the value of the restitution coefficient experimentally measured in a similar system of active vibrobots [2].

v) Subsequently, particle positions are updated over the remaining time interval, $\Delta t - t_{\rm coll}$.

The activated particle has the initial velocity $v_1(0) = v_0 = f_0/\gamma_0$, activity $n_1(0) = 1$ and coordinate $x_1(0) = -d/2$, while the particle at rest has zero initial velocity and activity $v_2(0) = n_2(0) = 0$ and coordinate $x_2(0) = d/2$. Figure S3 shows the dynamical evolution of the ensemble average computed over 10^3 stochastic trajectories obtained by numerically solving Eqs. (S4) using the EulerMaruyama scheme with a time step $\Delta t = 10^{-5}$, and simulation parameters $\tau_0^{-1} = 0.1$, $d = 1$ and $\kappa = 0.9$. Figure S3 illustrates the time evolution of the center of mass (a), the corresponding temporal evolution of the kinetic energy for $f_0 = 1$ (b), and the minimum kinetic energy (c) during the cooling process for various activity levels f_0. The qualitative behavior closely resembles that of the elastic collisions shown in Fig. 2 of the main text and the elastic collisions obtained through a WCA potential (Fig. S2). Notably, cooling is even more effective in this case: panels (a) and (b) reveal a reduced center-of-mass displacement and lower kinetic energy, while panel (c) shows that the range of activity values for which frictional cooling outperforms its Stokes counterpart is further extended. These results demonstrate that inelastic collisions enhance the cooling phenomenon presented in the main text.

SUPPLEMENTARY DISCUSSION 4. COOLED AND HEATED PHASES IN ACTIVE SYSTEMS WITH SELF-SUSTAINED FRICTIONAL COOLING.

Defining the effective temperature in active systems is ambiguous and open to various interpretations [3]. In this study, we identify whether the system is in the hot or cold phase by comparing the mode (the most probable speed) with the noise level. In this Supplementary Discussion, we demonstrate why the intuitive way of defining the temperature via the mean speed (or equivalently, via the mean kinetic energy) may be misleading to identify cooled and heated phases and may not properly reflect the features of an active system with self-sustained frictional cooling.

The mode speed represents the characteristic speed that most particles in the system tend to adopt, i.e. the low mode speed indicates that most particles in the sample are nearly stationary or cooled, in terms of their kinetic temperature, whereas a high mode speed implies that the particles move fast, since they are heated. In contrast, the mean speed is sensitive to the distribution's tails, which can be heavy in the cooled phase because of a small number of highly active particles that are statistically insignificant for the overall phase behavior. Consequently, measurements of the mean speed can occasionally yield values exceeding the typical noise level, even in the cooled phase, where the majority of particles move at speeds much lower than the noise level. This is confirmed in Fig. S4 (a) where we show a phase diagram in the plane of reduced activity f_0 and packing fraction Φ analogous to Fig. 4a in the main text, with

a color gradient of points denoting the mean particle speed (instead of the mode speed reported in the main text). Using the mean speed as a criterion may be misleading: in some cases, it gives the impression that a configuration in the cooled phase – which consists of a majority of almost stuck particles and a statistically irrelevant fraction of really fast particle – has a relatively large average kinetic energy (Fig. S4 (b)). However, this issue may appear only close to the phase boundary while in the bulk of the cooled phase both mean speed and mode speed provide the same information.

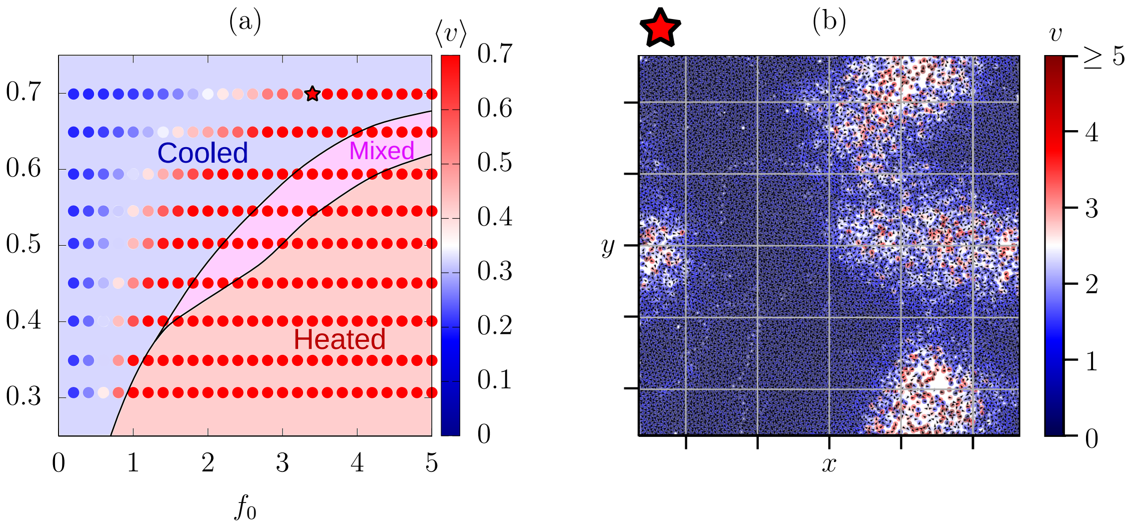

FIG. S4. **Kinetic phase diagram for mean particle speed.** Phase diagram in the plane of reduced activity f_0 and packing fraction Φ. Here, the color gradient denotes the mean particle speed $\langle v \rangle$ (points) rather than the mode speed v_m as in Fig. 4 (a) of the main text. Background colors are used to distinguish between different phases – blue (cooled), pink (mixed), and red (heated) as in Fig. 4 of the main text. (b) Snapshot of a cooled phase, where the color gradient denotes the particle speed (red for high and blue for low speeds). The red star above the snapshot indicates the corresponding parameters f_0 and Φ in the phase diagram (a). Despite the large value of the mean kinetic energy compared to the noise level, the snapshot shows a cooled configuration.

SUPPLEMENTARY DISCUSSION 5. SELF-SUSTAINED FRICTIONAL COOLING FOR PARTICLES GOVERNED BY STATIC FRICTION.

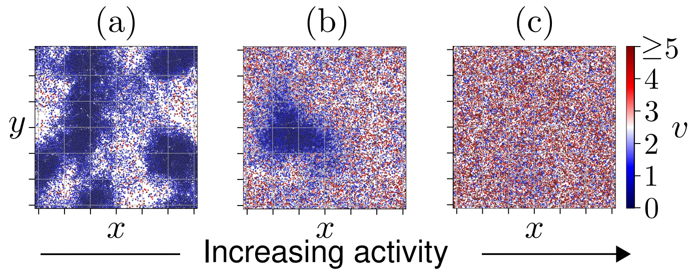

FIG. S5. **Effect of static dry friction.** (a) Cooled, (b) mixed and (c) heated phases for particle dynamics with the Tustin friction model (Eq. (S5)) which incorporates the effect of static friction. The dimensionless parameters of the simulations are $\tau_0^{-1} = 0.1$, $\epsilon_0 = 1$, $d = 1$, and $f_0 = 1.8$, 3.0, 4.0 for (a), (b) and (c), respectively.

In systems with dry friction, static friction refers to the phenomenon where it is more difficult to initiate motion than to maintain it once the object is already moving. In tribology, this effect is typically modeled using different friction coefficients for static and dynamic conditions, denoted by μ_s and μ_d respectively, with $\mu_s > \mu_d$. To check

the effect of static friction on the emerging phases, in this Supplementary Discussion, we consider the Tustin friction model [4]:

$$\dot{\mathbf{v}}_i(t) = -\boldsymbol{\sigma}(\mathbf{v}_i(t)) + \sqrt{\frac{2}{\tau_0}}\boldsymbol{\xi}_i(t) + f_0\mathbf{n}_i(t), \tag{S5a}$$

$$\dot{\mathbf{n}}_i(t) = -\frac{\mathbf{n}_i(t)}{\tau_0} + \sqrt{\frac{2}{\tau_0}}\boldsymbol{\eta}_i(t), \tag{S5b}$$

$$\boldsymbol{\sigma}(\mathbf{v}) = \hat{\mathbf{v}}\left(1 + \Delta_S e^{-|\mathbf{v}|/v_s}\right), \tag{S5c}$$

where $\hat{\mathbf{v}}$ denotes the normalized velocity vector. For the motion of polystyrene vibrobots on an acrylic plate studied in our experiments, the static friction coefficient μ_{s} ranges from 0.5 to 0.6, while the dynamic friction coefficient μ_{d} lies between 0.3 and 0.5. In our simulations, we adopt the largest possible value of $\Delta_S = (\mu_{\mathrm{s}} - \mu_{\mathrm{d}})/\mu_{\mathrm{d}} = 1$ within this range, and set $v_{\mathrm{s}} = 0.1$ as a representative velocity parameter in the Stribeck curve to model the crossover from static to dynamic friction. The results are presented in Fig. S5, where three snapshot configurations are shown for activity $f_0 = 1.8; 3.0; 4.0$, respectively. This study qualitatively confirms the presence of cooled (panel (a)), mixed (panel (b)), and heated phases (panel (c)), regardless of the inclusion of static friction. Indeed, static friction does not qualitatively affect the picture of self-sustained frictional cooling and it tends to promote the cooled phase. As a consequence, static friction is only responsible for a slight shift in the phase diagram (Fig. 4 of the main text).

[1] A. P. Antonov, L. Caprini, A. Ldov, C. Scholz, and H. Löwen, Phys. Rev. Lett. **133**, 198301 (2024).
[2] L. Caprini, D. Breoni, A. Ldov, C. Scholz, and H. Löwen, Commun. Phys. **7**, 343 (2024).
[3] L. Hecht, L. Caprini, H. Löwen, and B. Liebchen, J. Chem. Phys. **161**, 224904 (2024).
[4] L. Marton and B. Lantos, IEEE Trans. Ind. Electron. **54**, 511 (2007).
