# Supplementary material for: Self-sustained frictional cooling in active matter
Source: Nat Commun. 2025 Aug 6;16:7235. doi: 10.1038/s41467-025-62626-9 (PMC12328619; doi:10.1038/s41467-025-62626-9)
Supplement: Supplementary file 2 — Description of Additional Supplementary Files [file 41467_2025_62626_MOESM2_ESM.pdf]

Title: Supplementary Movie 1

Description: The steady-state motion of $N = 180$ active granular particles (vibrobots) with diameter $d = 15$ mm that are confined with a plate of diameter $D = 300$ mm. The direction of the active force is shown by white circular markers. The plate oscillates vertically at 110 Hz with amplitudes $A = 18.66 \pm 0.08$ μm (cooled phase), $A = 18.88 \pm 0.09$ μm (mixed phase), $A = 21.56 \pm 0.08$ μm (heated phase). In the cooled phase, particles are nearly immobile everywhere. In the mixed phase, particles inside the cluster stay immobile, while those outside are mobile. In the heated phase, all particles, including those in the cluster, move rapidly. The initial and final time instants correspond to those shown in Fig. 2 a-c of the main text.

Title: Supplementary Movie 2

Description: The steady-state motion of $N = 180$ active granular particles (vibrobots) with diameter $d = 15$ mm that are confined with a plate of diameter $D = 300$ mm. The direction of the active force is shown by white circular markers, and particle speed is represented by color: blue denotes slow particles, and red denotes fast ones. The parameters and particle trajectories are identical to those in Supplementary Movie 1.

Title: Supplementary Movie 3

Description: The initial motion of $N = 180$ active granular particles (vibrobots) with diameter $d = 15$ mm that are confined with a plate of diameter $D = 300$ mm. The direction of the active force is shown by white circular markers. The parameters are identical to those in Supplementary Movie 1. This video shows the initial phase of the experiment, preceding the steady state observed in Supplementary Movies 1 and 2.

Title: Supplementary Movie 4

Description: Numerical simulations of $N = 180$ active particles with diameter $d = 1$ that are confined with a soft plate of diameter $D = 20$. The direction of the active force is shown by arrows, and particle speed is represented by color: blue denotes slow particles, and red denotes fast ones. Other simulation parameters are $\tau_0^{-1} = 0.1$, $\epsilon_w/f_0 = 10^3$, $\epsilon_0/f_0 = 1$, and $f_0 = 0.4$, 1.0, 2.0 for cooled, mixed, and heated phase, respectively.
